# Diseases of the Hair and Scalp

**Published:** 1888-02

**Authors:** 


DISEASES OF THE HAIR AND SCALP. By Geo. Thos. Jackson, M. D. New York: E B. Treat, 1887. Price $2.75.

A classical treatise on a subject of daily interest and need. Its bibliography is very complete and covers the whole range of literature on this subject for the past twenty-five years. The arrangement is simple, and reference rendered quick and satisfactory by a full index. The chapter on hygiene of the hair and scalp contains advice which would benefit nearly every one, and its lessons and teachings, if duly impressed, would diminish baldness and dandruff. It is astonishing that the treatment of diseases of the hair, as of those of the skin also, should remain so complex, moldy in tone and spirit and difficult to carry out. The remedies and methods of their use should be simplified.

